# Discussions on Some Key Issues of Two-Dimensional Rotational Ultrasonic Combined Electro-Machining of Composite Materials

**DOI:** 10.3390/s23073741

**Published:** 2023-04-04

**Authors:** Wanwan Chen, Yongwei Zhu, Jing Li

**Affiliations:** 1 College of Mechanical Engineering, Yangzhou University, Yangzhou 225127, China; 2 College of Hydraulic Science and Engineering, Yangzhou University, Yangzhou 225127, China; 3 JITRI Institute of Precision Manufacturing, Nanjing 211800, China

**Keywords:** 2D-ultrasonic combined electro-machining, gap detection, machining experiments, parameters optimization

## Abstract

In order to improve the surface forming quality and machining efficiency of composite materials and reduce tool wear, a two-dimensional rotary ultrasonic combined electro-machining (2DRUEM) technology with low electrical conductivity and low current density was proposed in this study. Additionally, a gap detection unit of the machining system was designed with the integration of grinding force and gap current, and the average errors and maximum errors of the model were 5.61% and 12.08%, respectively, which were better than single detection. Furthermore, the machining parameters were optimally selected via NSGA-II, and the maximum machining surface roughness error was 5.9%, the maximum material removal rate error was 5.5%, and the maximum edge accuracy error was 8.9%, as established through experiments.

## 1. Introduction

With high strength, high hardness and excellent wear resistance, metal matrix composites can replace monolithic metal materials or traditional alloy materials in many specific aspects, and have been widely used in aviation, aerospace, transportation and other industries with important applications in the design and manufacture of new components, gradually moving towards an industrial scale [1,2,3,4]. However, the properties of the two matrix materials of composite materials differ greatly. Grinding machining (GM) is one of the final machining methods for finishing aluminum matrix composites, which cannot be ignored because of surface damage such as cracking, splitting, particle displacement, accumulation, and severe tool wear [5,6]. Electrical discharge machining (EDM) inevitably produces a recast layer on the surface from high-temperature melting and enhances the “shielding” effect of particles on the interpolar discharge, which reduces the efficiency of material removal [7,8]. Electrolysis machining (ECM), on the other hand, leaves a “skeleton” of reinforcing particles after the processing of conductive metal-based materials, which cannot improve the surface quality [9,10,11,12]. Wang et al. investigated the application of rotary ultrasound in machining CFRP materials from different perspectives and developed a material removal model, and their experimental results illustrated that in the case of ultrasonic compounding, while the cutting force was significantly reduced, the surface roughness increased [13,14,15,16]. Li et al. designed and modeled a new three-dimensional ultrasonic vibration platform with tunable characteristics. Their platform can realize a variety of vibration modes including one-dimensional (linear) and two-dimensional (in-plane or out-of-plane) vibrations [17]. Schubert [18], on the other hand, superimposed ultrasonic vibration on non-conductive ceramics with high-depth-to-diameter-ratio micro-hole discharge processing, where direct vibration of the workpiece or tool can increase the micro-hole depth by increasing the feed rate and reducing the inter-polar abnormal discharge state. Bhattacharyya et al. studied the effect of tool vibration on the clearance environment of electrochemical machining, which enhanced the electrode transient cavitation when the amplitude is increased, thus improving the material removal rate and machining accuracy [19,20]. Zhu Yongwei et al. studied ultrasonic electrolytic combined machining technology with a low-voltage passivating electrolyte for hard-to-machine metal materials such as cemented carbide, and the machining efficiency, accuracy, and surface quality were significantly improved, which can effectively solve the problems of machining hard and tough metal materials with complex shapes [21,22,23].

However, the material removal rate and machining accuracy are not satisfactory, no matter which machining method is used. Many scholars have proposed that high-frequency or high-amplitude ultrasonic vibration will further improve the electrolytic or machining performance. Even fewer studies have been focused on combined machining gap detection. Considering that there are many factors affecting machining efficiency and accuracy, and the interaction of factors is complex, the selection of machining parameters occupies a crucial position in the study of combined machining. In this study, we have determined the influence of gap detection on the effect of the combined machining process by comparing experiments with different processes of aluminum-based ceramic reinforced composite (SiCp/Al), according the material removal mechanism of 2DRUEM. The parameter selection model of machining process was established by NSGA-Ⅱ, and the material removal rate and surface roughness were studied using performance experiments under different machining parameters. The results basically realized the optimization goal of machining parameter selection.

## 2. Machining Principle of 2DRUEM

### 2.1. Analysis of Machining Mechanism

**Figure 1:**
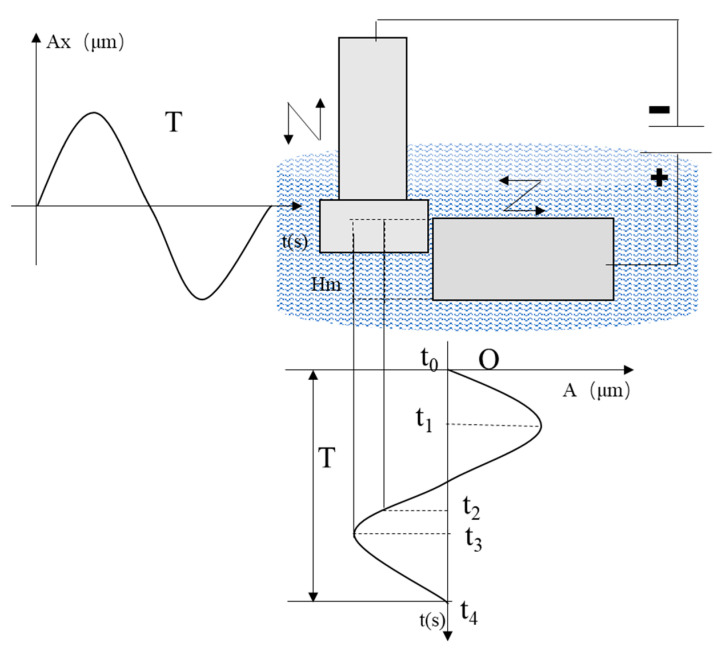
Schematic diagram of 2DRUEM machining process.

2DRUEM is an organic combination of ultrasonic vibration-assisted grinding (2DRUM), ECM and EDM. As the tool rotates, it vibrates in the axial direction and is fed in the X direction at a certain speed, while the workpiece vibrates tangentially to the direction of the tool feed. The workpiece is connected to the positive pole of the power supply and the diamond-coated conductive tool is connected to the negative pole of the power supply, and the inter-pole is filled with a low-conductivity passivated working fluid. The surface of the workpiece is removed by electrolytic dissolution, and the gas produced by the machining process is collected on the surface of the tool. When the gas film is formed and the vibration of the workpiece changes, the electric field strength between the poles becomes sufficiently large to break through the gas film, and the discharge occurs between the poles. When the workpiece vibration displacement brings the tool in contact with the workpiece, the abrasive grains grind the workpiece to remove the material, which increases the plastic shear domain of the brittle material due to the softening of the workpiece material by the ultrasonic vibration and the vibration prolongs the abrasive grinding path length, while the composite material macroscopically tends to exhibit more metallic properties. Material removal is influenced by the workpiece and the amplitude and frequency of vibration, feed rate, and rotational speed of the tool. Although the ultrasonic vibration effect increases the discharge probability and frequency, it is still negligible compared to electrolysis and grinding. The special form of material removal of composite materials leads to its unique surface generation mechanism. The workpiece has different forms of material removal with different vibration displacements of the workpiece, as shown in Figure 1. During the machining process, the electrolytic dissolution speed is mainly influenced by the workpiece amplitude $A_{x}$ and the initial machining gap $H(t_{0})$ of the tool electrode, while the axial vibration and rotational motion of the tool have less influence on the machining gap. During the workpiece feed cycle Δt, the machining gap H(t) mainly varies periodically with the workpiece vibration, which can be expressed as:

(1) $$H(t)=H(t_{0})+A_{x}\sin(\omega_{x}t)$$

When the microcurrent density is less than the threshold value, no electrical machining occurs between the poles. In contrast, when the microcurrent density is greater than the threshold value, there is an effective electrolytic critical gap α. It is assumed that there is an effective gap between the workpiece vibration balance and the tool. Additionally, when the voltage is less than 10 V for microfabrication, the interpolar gap is almost the same as that of RUEM and ECM, and the two-dimensional combined ultrasonic electro machining depth can be considered approximately equal to $\alpha +H_{e}$. The undeformed chip depth $H_{e}(t)$ during normal grinding is linearly related to *t* and the indentation depth $H_{p}(t)$ at the maximum coupling amplitude.

$$H_{e}(t)=H_{e}\frac{t}{\Delta t},\;H_{p}(t)=H_{p}\frac{t}{\Delta t}$$

where $H_{m}$ is the pressing depth at the maximum coupling amplitude.

There are two types of interpolar discharge machining—electrolytic discharge machining (ECDM) and EDM. When the machining gap is smaller than the thickness of the air film, the air film isolates the tool from the electrode insulation. When the inter-polar gap is smaller than the discharge gap value, which is about 10~20 μm, spark discharge occurs between the tool and the workpiece. During machining, air bubbles and cavitation bubbles precipitate from both poles collected on the tool surface under buoyancy and surface tension and form an air film. When the machining gap increases with the vibration of the workpiece and is larger than the thickness of the gas film (the thickness of the gas film is generally 0.1~0.2 mm), the gas film isolates the tool from the electrolyte and an electrolytic discharge occurs between the workpiece and the electrolyte. The electrolyte or tool with ultrasonic motion helps to form the air film faster and reduce the thickness of the air film, thus making discharge processing at a lower voltage possible.

### 2.2. Simulation of the Single Grain Impacts

**Figure 2:**
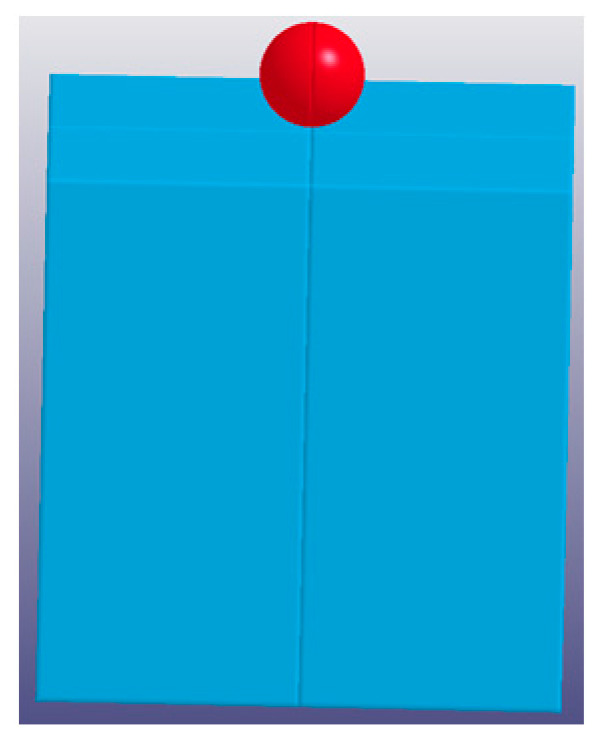
Grain shock simulation with finite element model.

In this paper, a finite element model was established directly from rotary sintered diamond grain tool head grinding, in accordance with the actual situation and boundary conditions of the vibrating tool head impacting the workpiece material, and the LS-dyna was applied to study the effects of grinding force and material removal. The workpiece material was silicon carbide, and the grinding grains were a diamond material. A face-to-face contact between the workpiece and the grinding grain was chosen, and a fixed constraint was attached to the bottom of the workpiece to limit the movement. The finite element model of this simulation was shown in Figure 2. The size of the grain was set to 5 μm in diameter, and the grain was given an initial velocity of 50 rad/s.

**Figure 3:**
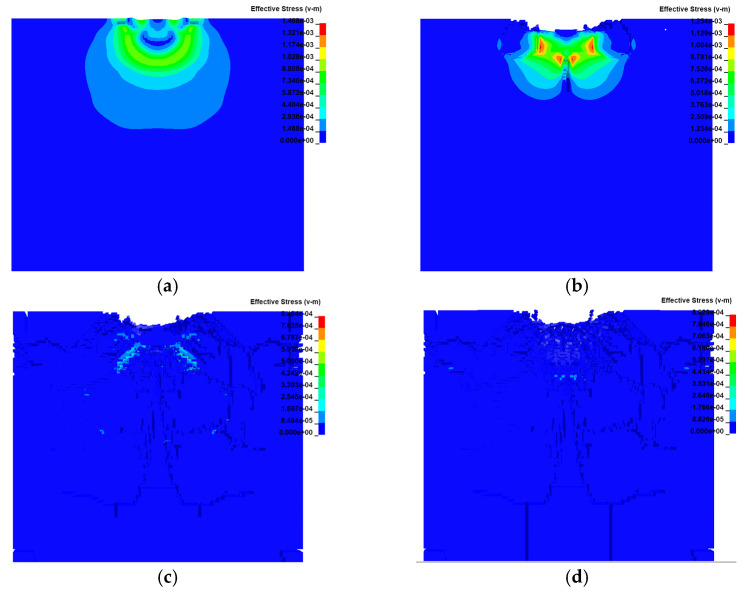
Microcrack extension process inside the material via perspective cloud maps at different times: (**a**) 0.2 s; (**b**) 0.4 s; (**c**) 1.2 s; (**d**) 2.1 s.

**Figure 4:**
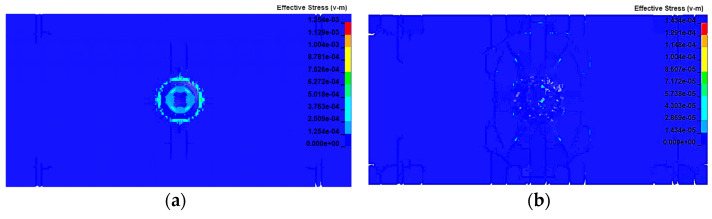
Cloud maps of the force conditions of grains’ impacts by the workpiece at different times: (**a**) 0.3 s; (**b**) 1.6 s.

Figure 3 presents cloud views of the simulation results. After the impact between the tool and the workpiece, a compressive stress wave was generated rapidly and propagated in the direction of the impact normal. At the beginning, the compressive stress on the contact surface increased sharply and the material started to deform. When the stress suffered by the material was greater than its own compressive strength, the material in the area below the contact surface broke, and the cracks inside the material due to the impact continued to grow in the impact direction. If the velocity continued to increase, microcracks appeared inside the material; the reason for these cracks was that the workpiece material produced elastic–plastic deformation after the impact, and the elastic deformation returned to cause high tensile stress within the material. The tensile strength limit of silicon carbide is much lower than the compressive strength limit, and the material was in a high strain rate state, meaning that the fracture toughness was greatly reduced, and the microcracks resulted from the generation of tensile stress below the elastic deformation region. Driven by the elastoplastic stress field, the microcracks gradually expanded along the edge of the elastoplastic deformation area on both sides; the greater the impact velocity of the abrasive grains, the greater the kinetic energy, the stronger the elastoplastic stress field generated inside the workpiece, and the greater the expansion distance. The microcracks continued to expand, driven by the internal stress field, and finally interconnected and penetrated, leading to the removal of large brittle fractures in the material. Since the energy required to form cracks was much greater than the energy required to drive crack expansion, the resistance to plowing and scratching of the abrasive grains on the workpiece decreased significantly when the abrasive grains impacted the workpiece to produce microcracks, resulting in a decreasing trend of tangential grinding force. Figure 4 shows the force situation of the spherical abrasive grain impacting the workpiece with multiple rotations in the rotary ultrasonic vibration processing. The maximum principal stress on the workpiece occurred when the workpiece material was in contact with the tool and started to break, and the maximum principal stress on the workpiece reached the maximum value with the increase in the tool amplitude. From the result, the maximum principal stress on the workpiece was 110.25 MPa.

### 2.3. Study of Material Removal during Processing

The surface generated by two-dimensional ultrasonic-assisted grinding machining was a three-dimensional morphology based on GM machining with a combination of single-particle two-dimensional ultrasonic fitting trajectory motion. The diamond abrasive grains embedded in the tool impacted and polished the workpiece surface, after which a new working surface was exposed. Electrolytic processing theory showed that during the effective period of power supply, the electrochemical anodic metal dissolution occurred on the surface of the workpiece due to electrolysis, with a thin and low-strength electrolytic passivation film being produced, which stopped the further electrolytic processing of the microcurrent. At the same time, the oxygen precipitated from the tool’s cathode surface was separated from the original surface and generated tiny bubbles rising under the action of buoyancy; some of the gas was mixed in the electrolyte to affect the conductivity, some of the gas gathered into a gas film covering the tool surface, and the rest escaped into the air. When the machining products and air film insulated the inter-pole and the X-direction ultrasonic vibration caused the inter-pole gap to reach the critical value of an electric spark, the insulating compartment was penetrated and a discharge channel was formed, resulting in electric spark discharge and etching of the workpiece surface via vaporization and melting.

**Figure 5:**
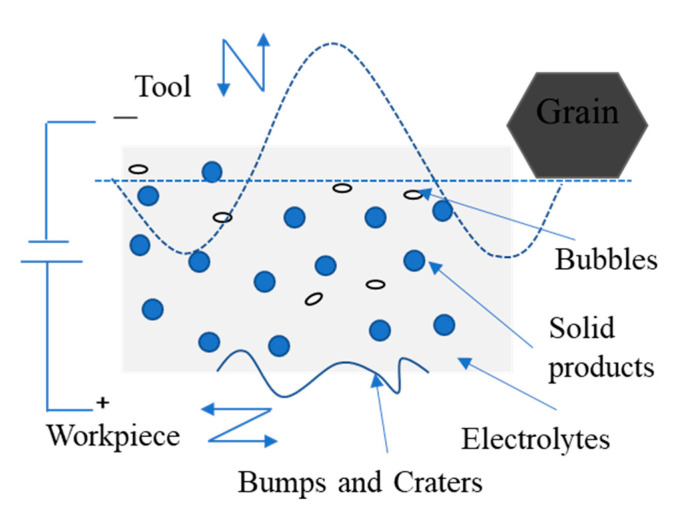
Schematic diagram of the 2RUEM material removal principle.

In the contact area of 2DRUEM, the abrasive grains on the tool abraded and scraped the workpiece surface via ultrasonic vibration and axial rotation; the axial ultrasonic vibration increased the contact arc length, thus increasing the electrolytic processing area, and the machining products were instantly stripped from the workpiece surface, some of which were flushed out of the work area by the electrolyte between poles and the rest were pressed on the tool surface. Along with the radial ultrasonic vibration grinding and flattening, the surface bumps of the workpiece can be reduced, and the local electrolysis and EDM can be reduced, which can affect the flatness of the surface, while the gap between the poles can be changed to achieve small gap electrolysis and provide conditions for EDM formation. Rotary ultrasonic grinding and EDM worked together on the surface of the workpiece, removing the passivation film produced by electrolysis, and at the same time exposing a new metal surface to activate it, which can improve the efficiency of material removal, as simply shown in Figure 5.

In the power interval region, the electric field between the two poles rapidly decreased to 0, the discharge channel disappeared, leading to the disappearance of electrolysis, the disturbance of rotating ultrasonic vibration caused the machining products in the interpolar region to be discharged even further, and the electrolyte was renewed, combined with the cooling of the grinding contact area. The gap between the poles of the machining process changed periodically under the action of ultrasonics so that the purpose of accelerating the etching of materials with small gaps and renewing the electrolyte with large gaps could be achieved, and the surface quality and dimensional accuracy of the workpiece could be improved compared with that of ECM. The different roles of the two types of ultrasonics in the complex mechanism work together to achieve the goal of an efficient and high-quality removal process of the combined machining. In rotary ultrasonic machining, the motion trajectory of the abrasive grains on the side of the tool is sinusoidal due to ultrasonic vibration. The total length of the abrasive grains scratching the surface of the workpiece bore wall was longer in rotary ultrasonic machining compared to normal grinding. At the same time, a mechanism of superposition of abrasive trajectories was formed—that is, the motion trajectories of adjacent abrasive grains were be superimposed due to the ultrasonic vibration, which is favorable for the reduction in surface roughness.

These three machining processes are an organic whole of interaction and efficient synthesis, which can improve the machining speed and achieve better surface quality.

## 3. Study of Gap Status of Machining Process

### 3.1. Multi-Sensor Information Fusion Detection

**Figure 6:**
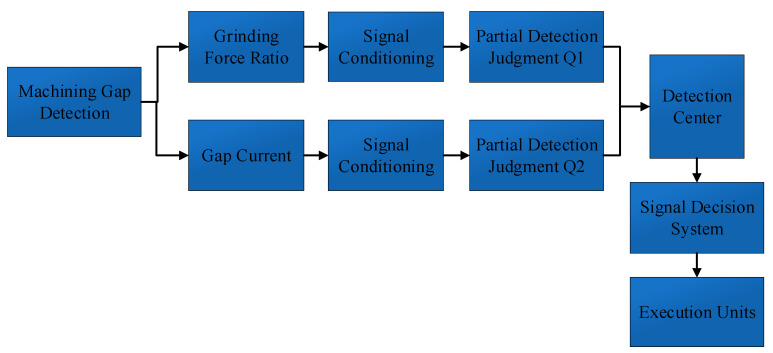
Fusion model of machining gap detection.

According to characteristics of 2DRUEM, grinding processing occupies the main position, along with ultrasonic vibration-assisted processing and electrolysis-optimized processing effect, and this paper proposes a method of target identification based on the information fusion model of multi-sensor measurement, as shown in Figure 6. This method fuses the information detected by two different types of sensors with grinding force and machining current, thus avoiding the limitation of single measurement information and realizing multi-system online inspection.

When the workpiece was shorted, the surface texture became rough and ripples appeared at the edges, and in serious cases, the surface of the workpiece produced coated areas in the form of alluvial deposits, which led to frequent contact between the workpiece and the tool head, as well as an increase in the grinding force ratio $F_{n}/F_{t}$ and a rise in the discharge and short-circuit rate, and the grinding force and current changes had opposite trends, so that the two could be used as collection quantities to complement each other and avoid decision-making errors.

The force measuring instrument and short-circuit status detection unit were used to collect the grinding force ratio $F_{n}/F_{t}$ and the discharge short-circuit rate through dual channels, and then the control system could classify and count the data collected during the period of time to make a comprehensive judgment of the machining status and to decide on the control steps.

The optimization of combined machining parameters was composed of two parts: firstly, a model was established to represent the relationship between machining parameters and process targets, and then the objective function was constructed according to the model with the optimization algorithm adopted to obtain the optimal combination of parameters.

### 3.2. Control Process

According to the control output results of ultrasonic amplitude, grinding feed rate and electrolytic power switch control parameters on the different roles of the machining state, the control process was graded based on the detection results.

Through analysis, the fuzzy logic operation unit was the core of the whole control system, and the module was responsible for whether the output parameters were adjusted and the size of the adjustment amount, so the control system also needed to consider the adaptive capability of the control system with the algorithm requirements. The combined processing control strategy was controlled in real time, being divided into phases. $\sigma_{p}$ was the real-time grinding force ratio, and $\sigma_{p0}$, $\sigma_{p1}$, and $\sigma_{p2}$ were the grinding force ratio thresholds set for each phase.

(1) When $\sigma_{p0}∠\sigma_{p}\leq \sigma_{p1}$, the ultrasonic amplitude was controlled;
(2) When $\sigma_{p1}∠\sigma_{p}∠\sigma_{p2}$, the feed rate of the workpiece was controlled;
(3) When $\sigma_{p2}\leq \sigma_{p}$, we stopped feeding the workpiece or moved it back, and controlled the electrolytic power supply;
(4) When $\sigma_{p}\leq \sigma_{p0}$, the machining parameters remained unchanged, but when the gap current was greater than the threshold set by the short-circuit current, the power supply was adjusted. When the grinding force ratio and the gap current returned to normal, the power supply started again.

**Figure 7:**
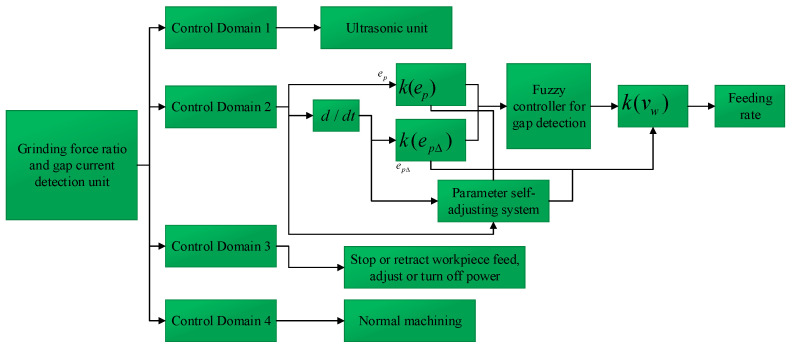
Control flow diagram.

The control flow diagram is shown in Figure 7. During the machining process, if the grinding force ratio signal and current signal were within the control range of the machining system, they were sent to the fuzzy control unit, and the controller analyzed and processed them to adjust the feed rate and power supply. In the control process, the size $e_{p}$ of the deviation $\sigma_{p}$ from the critical value $\sigma_{p1}$ was used as one of the reference conditions to determine the adjustment amount. In order to control the stability of the system and reduce the oscillation and overshoot of the process control, $e_{p}{}_{\Delta }$ was the rate of change $e_{p}$ of the control, also used as another reference to control the adjustment amount. We compared the results with the set critical grinding force threshold based on the real-time detection grinding force ratio $\sigma_{p}$, and when the real-time detection grinding force ratio was greater than the set value, the workpiece feed rate was controlled in order to avoid short-circuiting.

### 3.3. Information Fusion Algorithm

In this paper, an information fusion method based on the PCA method was introduced to complete the feature selection and information fusion. Combined with the specific algorithm of PCA method, the specific implementation process of the feature selection method applied to the information fusion system was designed. Firstly, the feature matrix K for multi-sensor signal extraction was constructed through sample experiments, the Z-score normalization of the data was completed, the covariance matrix $C_{p\times p}$ was calculated, ranking of the feature values from large to small and the sorting between the corresponding feature vectors was completed, and the variance contribution rate α of the integrated feature dimension vectors was established. Different variance contribution rate thresholds were selected for the screening of the feature vectors, and the sorted feature vectors after screening constituted the dimension reduction matrix, which was compared with the original feature matrix to obtain the integrated feature matrix after dimensionality reduction.

**Figure 8:**
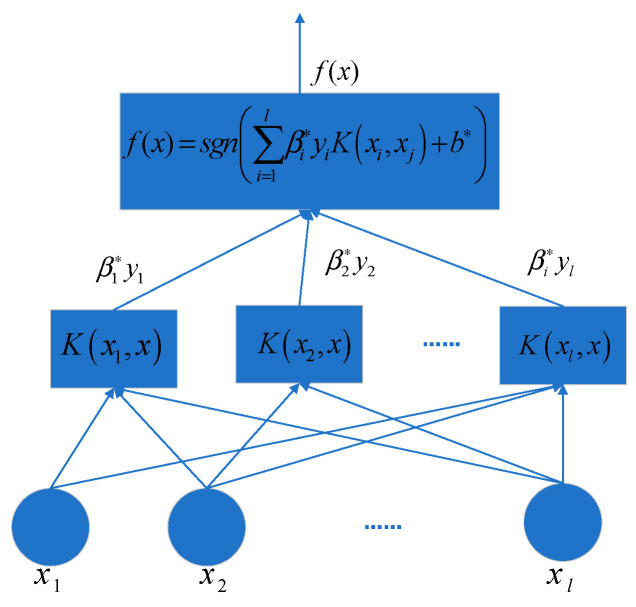
Basic structure of SVM.

Assuming that there were K features extracted by the fusion system and n samples, the dimension vector corresponding to the i feature is $S_{j}$, the sample vector corresponding to the *j* sample is $T_{j}$, and the mean $\bar{x_{i}}$ and variance $Var(x_{i})$ of the feature dimension vectors are calculated and the covariance could be solved.

(2) $$K=\left(\begin{array}{ccccc} 1 & x_{11} & x_{12} & \cdots & x_{k}{}_{1}\\ 1 & x_{21} & x_{22} & \cdots & x_{k2}\\ \vdots & \vdots & \vdots & \vdots & \vdots \\ 1 & x_{1n} & x_{2n} & \vdots & x_{kn} \end{array}\right)$$

(3) $$S_{i}=\left(x_{i1},x_{i2},\cdots ,x_{in}\right),i=1,2,\cdots k$$

(4) $$Cov\left(S_{a},S_{b}\right)=\frac{1}{n-1}\sum_{j=1}^{n}\left(x_{aj}-\bar{x_{a}}\right)\left(x_{bj}-\bar{x_{b}}\right),a,b\in \forall \left\{1,2,\cdots k\right\}$$

(5) $$C_{p\times p}=\left\{c_{ij}|c_{ij}=Cov\left(X_{S-\dim}\left(i\right),X_{S-\dim}\left(j\right)\right),X_{S-\dim}\left(i\right)=S_{j}\right\},i,j=1,2,\cdots k$$

where $Cov\left(S_{a},S_{b}\right)=Cov\left(S_{b},S_{a}\right)$, and the variance contribution rate is $\alpha_{i}$

(6) $$\alpha_{i}=\lambda_{i}/\sum_{i}^{k}\lambda_{i}$$

where $\lambda_{i}$ is the eigenvalue of solving the covariance matrix. Then, the support vector machine algorithm (SVM) was used to identify and select parameters, and the basic structure is presented in Figure 8.

For regression and the fitting problem, assuming random samples with an unknown function set as the input vectos, the expected value $G={\left\{\left(x_{i},p_{i}\right)\right\}}^{n}$, and *n* is the sample size. A linear function of the support vector machine was constructed in high-dimensional feature space:

(7) $$y(x)=w^{T}\psi (x)+a$$

where w,x∈R,a∈R, ψ(x) is the sample vector of the original space, and after the nonlinear mapping of the obtained high-dimensional feature space vector, the weight vector w and the deviation a can be obtained via minimizing.

(8) $$R(D)=D\frac{1}{n}\sum_{i=1}^{n}L\left(p_{i},y_{i}\right)+\frac{1}{2}{\left\|w\right\|}^{2}$$

(9) $$L_{\partial }(p,y)=\left\{\begin{array}{c} \left|p-y\right|-\partial ,µ\\ 0, \end{array}\right.\begin{array}{c} \left|p-y(x)\right|\geq \partial \\ others \end{array}$$

where L(p,y) refers to the ∂-insensitive loss function, which indicates that the sample data with errors ∂ below the threshold would not be penalized (i.e., discarded). $D\frac{1}{n}\sum_{i=1}^{n}L\left(p_{i},y_{i}\right)$ is the empirical risk, $\frac{1}{2}{\left\|w\right\|}^{2}$ indicates the smoothness of the function, and ∂ and *D* are constant parameters set in advance, with ∂ denoting the prediction accuracy of the support vector machine and *D* being the penalty factor. In order to ensure that the above optimization problem would have a solution, slack variables needed to be introduced, and the optimization equation became:

(10) $$\min R(w,a,\xi ,\hat{\xi })=\frac{1}{2}{\left\|w\right\|}^{2}+D\sum_{i=1}^{n}\left(\xi +{\hat{\xi }}_{i}\right)$$

The approximate equation in the default was:

(11) $$\left\{\begin{array}{c} p_{i}-y_{i}\leq \xi_{i}+\partial \\ y_{i}-p_{i}\leq {\hat{\xi }}_{i}+\partial \\ \xi_{i},{\hat{\xi }}_{i}\geq 0,1,2,\cdots ,n \end{array}\right.$$

Under the defined constraint equation, the Lagrange equation and the Lagrange multiplier were introduced to solve the above optimization equation, where w, a, $\xi_{i}$, ${\hat{\xi }}_{i}$ are the original variables and $\alpha_{i}\geq 0$ are the pairwise variables. By solving the partial derivatives of the original variables in Equation (11), the pairwise problem can be converted into a problem of solving the maximum of the following equation:

(12) $$\max\left\{\sum_{i=1}^{n}d_{i}\left(\alpha_{i}-\alpha_{i}^{\prime }\right)-\partial \sum_{i=1}^{n}\left(\alpha_{i}-\alpha_{i}^{\prime }\right)-\frac{1}{2}\sum_{i=1}^{n}\sum_{i=1}^{n}\left(\alpha_{i}-\alpha_{i}^{\prime }\right)\left(\alpha_{j}-\alpha_{j}^{\prime }\right)K\left(x_{i},x_{j}\right)\right\}$$

(13) $$\left\{\begin{array}{c} \sum_{i=1}^{n}\left(\alpha_{i}-\alpha_{i}^{\prime }\right)=0\\ 0\leq \alpha_{i}\leq D\\ 0\leq \alpha_{i}^{\prime }\leq D \end{array}\right.$$

where $K\left(x_{i},x_{j}\right)=\psi^{T}\left(x_{i}\right)\psi \left(x_{j}\right)$ is the inner product kernel function of the original sample set x satisfying Mercer’s theorem, and after solving the pairwise problem, all the weights can be expressed by *n* non-zero Lagrange multipliers $\alpha_{i}$, $\alpha_{i}^{\prime }$, and the corresponding learning vectors $x_{i}$. Finally, the optimal decision function of the support vector machine can be expressed as:

(14) $$y(x)=\sum_{i=1}^{n}\left(\alpha_{i}-\alpha_{i}^{\prime }\right)K\left(x,x_{i}\right)+a$$

## 4. Optimization Study of Machining Parameters

### 4.1. Establishment of Optimization Model

The selection of machining parameters in the combined machining process should not only ensure the individual machining process indexes but also concern the mutual constraints between the process indexes, so traditional parameter selection methods cannot be applied directly to the actual machining. In the combined machining process, peak current, machining voltage, spindle speed, machining time, machining depth, machining area, electrode shape, electrode material, working fluid, ultrasonic frequency, ultrasonic amplitude, electrode speed, abrasive particle size, feed rate and many other influencing parameters are related to the combined machining process. According to the single-factor experiments, this thesis focused only on the optimization of parameters of ultrasonic amplitude ($A_{x}$, $A_{z}$), gap voltage (U), feed rate ($v_{w}$) and rotational speed ($v_{s}$) for limited conditions. Additionally, the main parameters reflecting the machining effects were the material removal rate, surface roughness, and the average roughness of the contour edges.

(15) $$\left\{\begin{array}{c} v_{s}{}_{\min}\leq v_{s}\leq v_{s}{}_{\max}\\ U_{\min}\leq U\leq U_{\max}\\ A_{z}{}_{\min}\leq A_{z}\leq A_{z}{}_{\max}\\ A_{x}{}_{\min}\leq A_{x}\leq A_{x}{}_{\max}\\ v_{w}{}_{\min}\leq v_{w}\leq v_{w}{}_{\max} \end{array}\right.$$

(16) $$\begin{array}{l} \min F_{i}(X)\;i=1,2\cdots n\\ s.t.\left\{\begin{array}{c} MRR(X)\in \left[MRR\right]\\ \begin{array}{l} R_{a}(X)\in \left[R_{a}\right]\\ R_{e}(X)\in \left[R_{e}\right] \end{array}\\ X=\left(x_{1},x_{2},\cdots \right)\in K^{m} \end{array}\right.\\ X=\left(v_{s},v_{w},U,A_{x},A_{z}\right) \end{array}$$

where $F_{i}(X)$ is the objective function, N is the number of objective functions, MRR(X), $R_{a}(X)$, and $R_{e}(X)$ are the constraint functions, X is the decision vector, *m* is the number of decision variables, and the multi-objective optimization function is as follows:

(17) $$\min F_{i}(X)=\left[\begin{array}{c} f_{1}(X)=-MRR\\ f_{2}(X)=R_{a}\\ f_{3}(X)=R_{e} \end{array}\right]$$

It can be found that the optimization of machining parameters is a typical multi-objective optimization problem regarding the efficiency and quality of machining processing, and the traditional single-objective genetic algorithm cannot be used. In the process of single-objective optimization, when the optimization process stops, the global maximum or minimum solution is obtained, i.e., the optimal solution, which is better than all other solutions.

However, when there are multiple objectives, each objective is constrained to each other and there are conflicting relationships, so the optimal solution obtained after optimization for a certain objective function may not fit other objective functions, and may even be worse, so it is difficult to obtain an optimal solution that can satisfy multiple objectives at the same time.

**Figure 9:**
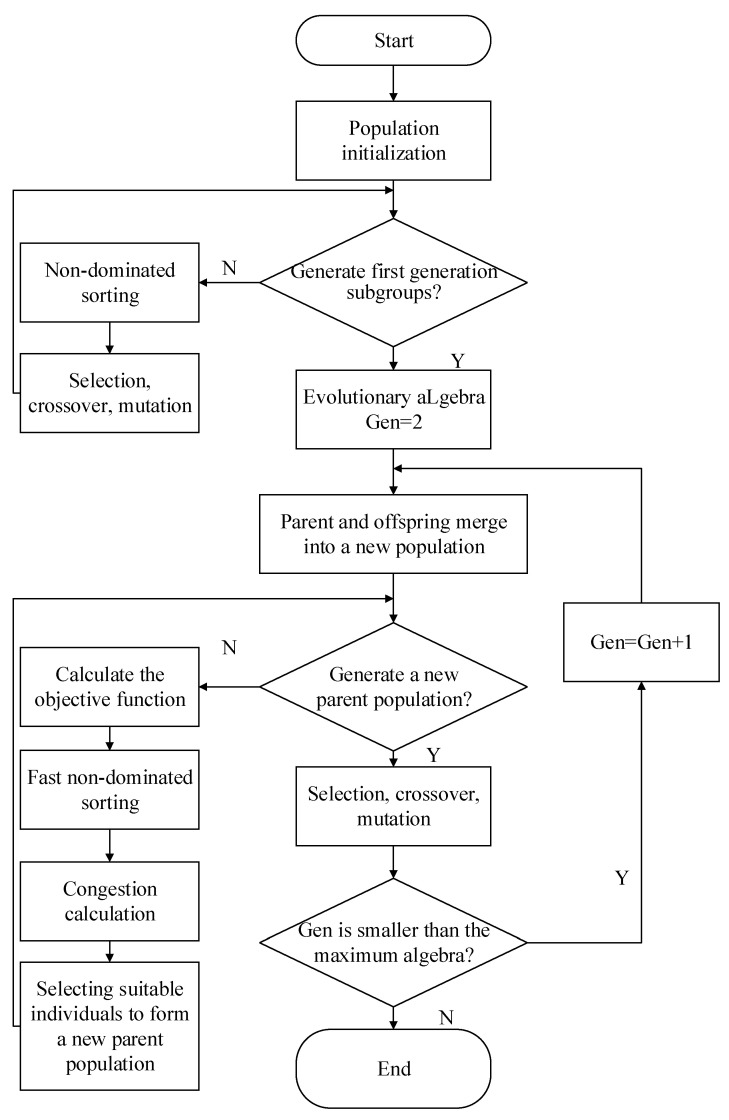
NSGA-II algorithm flow chart.

The NSGA-II algorithm is based on fast non-dominated sorting and an elite retention strategy, which retains the parent’s excellent genes to perform non-dominated sorting together with the offspring, and introduces crowding degree and crowding degree comparison operators, and finally the Pareto optimal solution can be obtained, composed of a set of multiple optimal process parameters, and the algorithm flow chart is shown in Figure 9.

The orthogonal experiments were conducted with 60% SiCp/Al composites, the tool electrode was a 6 mm diameter diamond with 400 mesh abrasive density, and the electrolyte was 5% NaNO_3_ with a 400 mesh silicon carbide. The orthogonal experimental process parameters are listed in Table 1 and the orthogonal experimental results are given in Table 2.

In order to evaluate the influence degree of machining parameters, the main effect factor analysis method was used, and the results are listed in the table. In statistics, the factor effect was defined as the difference between the two extreme values obtained by the factor, which was an absolute value. For comparison purposes, the relative factor effect was defined in this paper as follows:

(18) $$ERR_{i}=\frac{ER_{i}}{\sum_{j=1}^{n}E_{j}}$$

where $ERR_{i}$ is the first factor effect, *n* is the total number of factors, and only five four-level factors were considered in this section. The relative factor effects are listed in Table 3.

### 4.2. Study on Optimization Algorithms

Three-objective process optimization may face difficulties related to the degradation of population diversity and early convergence in multi-objective optimization, which may lead to obtaining a local optimal solution for the algorithm, especially for high-dimensional objectives.

In multi-objective studies, most of the literature has been devoted to the improvement of the speed of the algorithm, and its diversity has rarely been evaluated, but in practical engineering problems, it is more important to find the appropriate and effective process optimization parameters. The three-objective optimization model maximizes MRR and minimizes $R_{a}$, $R_{e}$ as the primary objective to optimize the process parameters. In the NSGA-II algorithm, all of the objective functions sought to obtain the minimum value, taking the inverse of the setting; therefore, the objective functions of the three-objective optimization were as follows:

(1) Objective1 = $G_{Ra}$;
(2) Objective2 = GRe;
(3) Objective3 = $-G_{MRR}$.

In order to verify the validity of the Pareto frontier obtained via the three-objective optimization, three sets of experiments comparing the relevant parameters of NSGA-II were used, the first set being:

(a) Population size (pop) = 200;
(b) Number of population generations (gen) = 300;
(c) Variance probability (mu) = 0.3;
(d) Crossover probability (cr) = 0.8.

**Figure 10:**
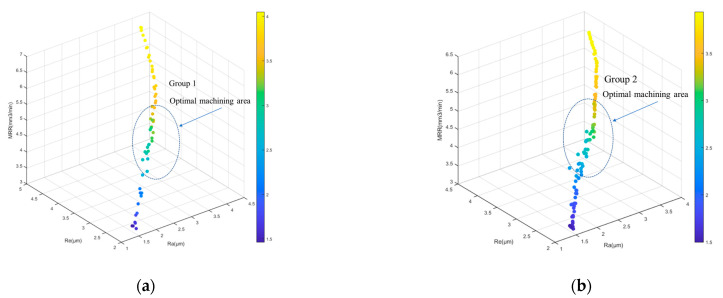
Multi-objective optimization frontier: (**a**) Group 1; (**b**) Group 2.

The second group increased the population size and the number of population generations, and the other two parameters did not change; the parameters that changed were (a) pop = 300 and (b) gen = 500. The results of the optimization model showed that the process parameters were optimized in the direction that maximized MRR and minimized $R_{a}$, $R_{e}$, which was similar to the two-objective optimization, due to the fact that NSGA-II allowed the coexistence of all non-dominated frontiers. The difference between the fronts of the two groups of experiments was not significant, and the Pareto fronts marked by the blue elliptical boxes in the figures represent the final results of the integrated three-objective optimization, while the machining parameters are presented in Table 4. The optimization results are shown in Table 5, and Figure 10 presents the multi-objective optimization frontier. The mean value of surface roughness was 2.58 μm, that of edge roughness was 3.57 μm, and the mean value of the material removal rate was 2.01 mm^3^/min, according to the optimized parameters. 

## 5. Experimental Studies

**Figure 11:**
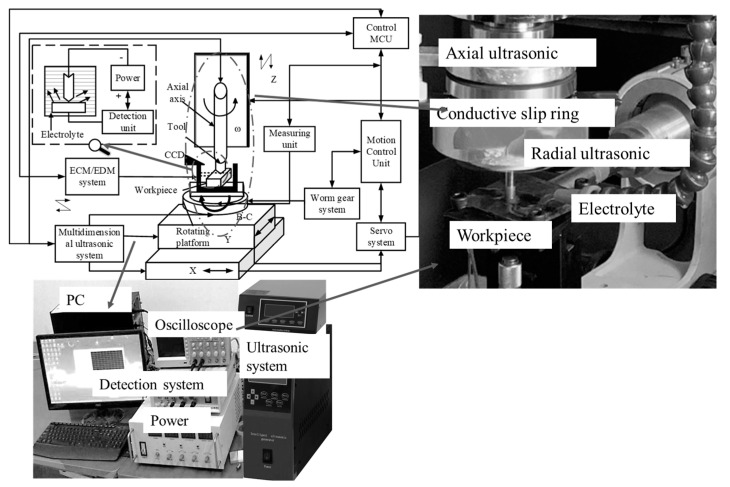
2DRUEM experimental system.

The principle of 2DRUEM machining was analyzed and explained in a previous paper. In order to confirm that the method can improve the machining quality of the combined electro-machining system, and that the gap detection and machining parameter optimization can both improve the machining system and enhance the machining quality, comparative experiments were designed to judge the comprehensive impact of the designed system on the machining effect by comparing the experimental results. The combined machining system used in this study was modified from the previous experimental setup, as shown in Figure 11. The experimental setup mainly consisted of the ultrasonic vibration system, the motion system, the control system, the electrochemical machining system and the electrolyte supply system, along with the machining status monitoring system and some other systems.

The positive electrolytic power supply was connected to the workpiece, and the negative electrode was connected to the negative tool head cathode, while the workpiece was insulated from the machine tool as a whole during the machining process. The spindle was fed up and down in the Z direction, and the table plane was fed in the X and Y directions. All of the X, Y and Z axial feeding movements were driven by the corresponding servo motors and were controlled centrally by the CNC system. The two-direction ultrasonic system was adopted, using digital ultrasonic generators with automatic frequency tracking and feedback adjustment. The ultrasonic frequency was 16 kHz~24 kHz, and the amplitude could be adjusted in real time according to the actual needs regarding power, voltage and current. The basic parameters of the experimental procedure are shown in Table 6.

### 5.1. Gap Status Detection Experiments

#### 5.1.1. Grinding Force and Current Detection Experiments

**Figure 12:**
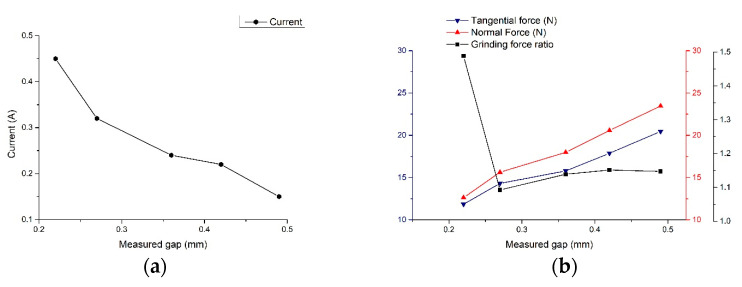
Relationship between the current and grinding force with a machining gap:(**a**) current; (**b**) grinding force.

In order to study the feasibility of the online inspection scheme for the machining gap based on the fusion model of the grinding force ratio and current, experimental studies on the relationship between the grinding force and gap current with a machining gap were conducted using the machining system, and the experimental results are shown in Figure 12.

According to the machining results, it can be seen that the gap current gradually increased as the machining gap decreased, and the grinding force increased as the machining gap increased. Further analysis indicated that the current increased faster when the machining gap was smaller, and the normal force increased more than the tangential force.

**Figure 13:**
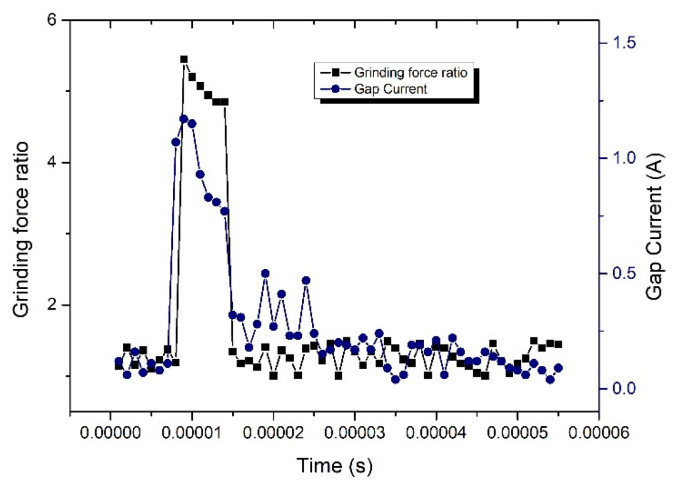
Change in current and grinding force ratio before and after the occurrence of short-circuiting.

**Figure 14:**
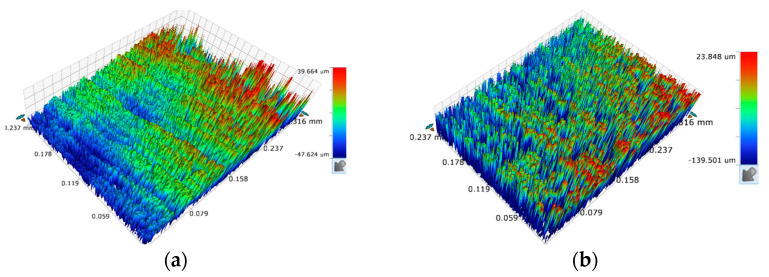
Machining results with and without gap detection: (**a**) without gap detection; (**b**) with gap detection.

When a short-circuit occurred, the current instantaneously became higher and the grinding force ratio instantaneously increased, as shown in Figure 13, and the sudden changes in current and grinding force were almost synchronized, which further demonstrated that the use of grinding force and current detection as a control to judge the gap decision. Figure 14 shows the 3D microscopic surface of the workpiece with and without gap inspection, and it can be seen that the machining quality was worse, and the grinding marks on the machined surface are serious and uneven without gap inspection.

To further verify the accuracy of the multi-sensor fusion gap detection system designed in this paper, the experiment carried out with sample was repeated three times, and the features extracted and processed in two of the experiments were selected as the sample set with the corresponding actual measured gap values. Training validation was performed via the use of the information fusion method algorithm to check whether its prediction accuracy for the processing gap meets the requirements.

**Figure 15:**
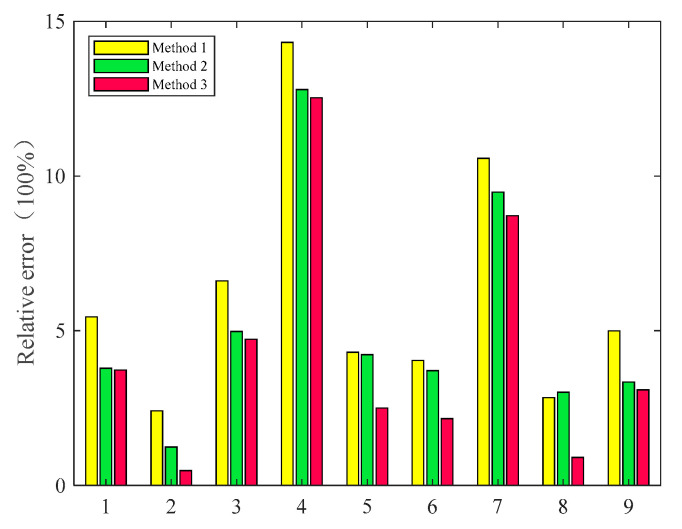
Relative error of test samples.

The relative errors of the nine test samples are shown in Figure 15, where Method 1 represents the values predicted using only the current value, Method 2 represents the values predicted using the grinding force ratio, and Method 3 represents the values predicted using the fusion algorithm. The average relative errors of the three methods were 8.77%, 9.71%, and 5.61%, respectively, and their maximum relative errors were 12.8%, 14.75%, and 12.08%, respectively. Thus, the performance of the fusion model can be evaluated as being better than that of the single sensor, both in terms of relative error and in terms of average relative error. The maximum error of the fusion model was reduced by 0.92% relative to Method 1, and in comparison to Method 2, the maximum error of the fusion model was reduced by 3.67%, both of which represent relatively significant improvements, and this improvement did not sacrifice the average relative error as an evaluation metric. Therefore, for the multivariate nonlinear gap detection of electro-machining processes, the use of the PCA-based support vector machine fusion algorithm can improve the prediction accuracy of the model with relative advantages over the use of a single sensor, which can improve the prediction accuracy of the model.

#### 5.1.2. Influence of Gap Detection on Machining Effects

The comprehensive impact of the designed gap detection on the machining effect was analyzed through experiments based on the presented combined machining system, mainly on the impact on the machining quality.

The previous analysis describes the detection principle of the combined machining process by means of gap detection. Theoretically, this method can avoid the short-circuiting of the tool electrode and the surface of the workpiece caused by a high current during the machining process, and thus improve the quality of the combined electro-machined workpiece. In order to prove that the method can play a beneficial role in protecting against short-circuiting in combined electric machining while also improving the machining quality, two groups of comparative experiments were designed: one group of experiments introduced the gap short-circuit detection method to achieve short-circuit protection for combined electric machining, and the other group of experiments did not. The comparison of the two groups of experimental results was used to determine the impact of the gap detection short-circuit method on machining quality.

In the process of combined machining, in order to reduce the discharge of machining debris and reduce the occurrence of short-circuiting in the machining circuit, a lower feed rate is usually used, which increases the input cost of the electrolyte on the one hand but reduces the actual machining efficiency on the other. However, with the gap detection method, when the control system detected a short-circuit, it was able to send a signal back to the power supply, activating the ultrasonic and servo systems until such a time that the electrode short-circuit ended and the tool electrode started feeding again, thus making the machining process continuous and stable. Therefore, the impact of the proposed detection method on the stability of machining of composite electrodes can be illustrated by increasing the feed rate and conducting a comparative experiment between the two cases of short-circuit detection with and without a gap.

**Figure 16:**
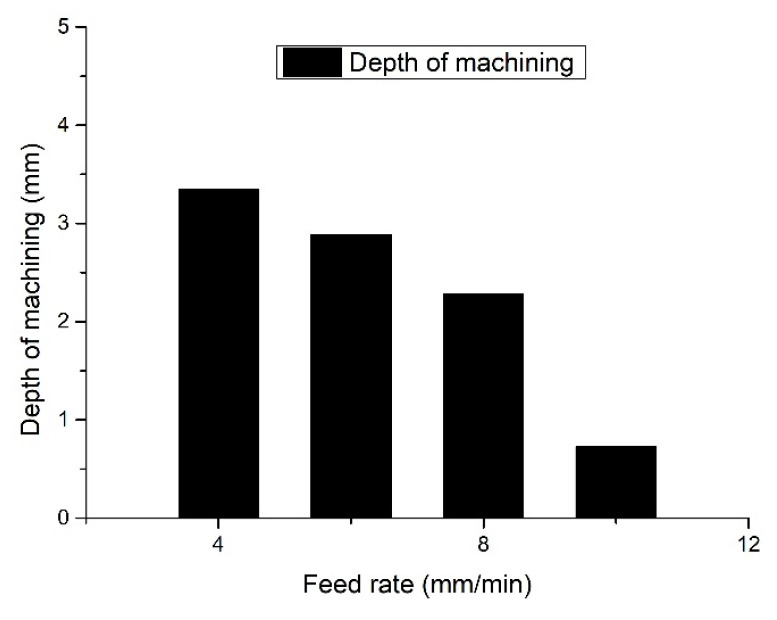
Machining depth with different feed rates without detection.

The basic parameters used in this experiment were the same as in a previous paper, and the corresponding feed rates of the tool electrodes were 4 mm/min, 6 mm/min, 8 mm/min and 10 mm/min. The experimental results are shown in Figure 16. When the machining was stopped automatically after a short-circuit was detected without a detection system, a short-circuit occurred after 3.42 mm of machining at a feed rate of 4 mm/min; a short-circuit occurred after 2.89 mm of machining at a feed rate of 6 mm/min; and a short-circuit occurred after 2.24 mm of machining at a feed rate of 8 mm/min. When the feed rate was increased to 10 mm/min, the short-circuit occurred when the machining was carried out to 0.83 mm and the machining was forced to stop. From the above experiment, it can be seen that as the feeding speed of the tool electrode increases, the time interval from the start of machining to the short-circuit between the tool electrode and the workpiece is shorter and the machining depth is shallower. Additionally, when there was gap detection for a short-circuit in the machining process of combined machining at different feed speeds, the short-circuit detection protection was set in that condition. The machining process was found to be continuous for all of the same feed speed conditions. Even with the increase in the feed speed, the whole machining process can be still ensured, which was completely different from the machining result with no gap short-circuit detection.

**Figure 17:**
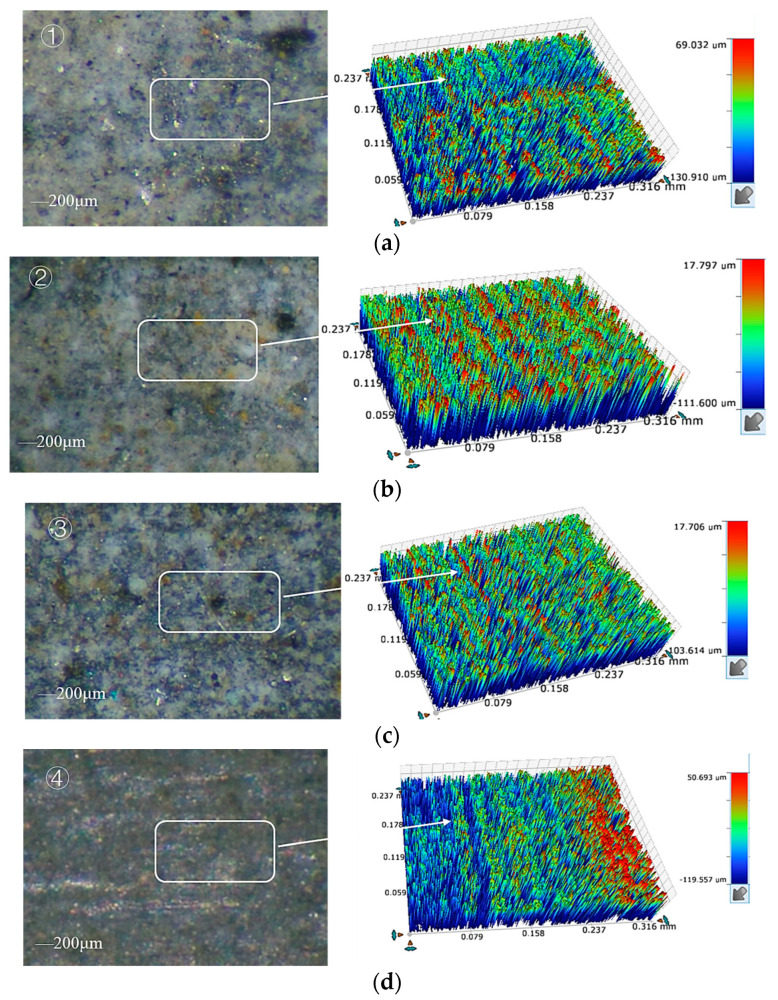
(**a**) 3 V; (**b**) 4 V; (**c**) 5 V; (**d**) 6 V.

**Figure 18:**
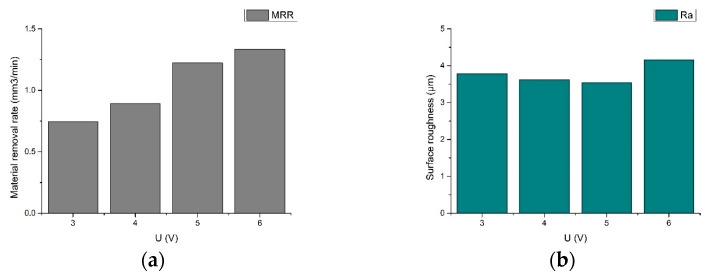
Material removal rate and surface roughness variation with machining voltage: (**a**) material removal rate variation; (**b**) surface roughness variation.

In a combined electric machining system, the effect of voltage on machining efficiency plays an important role. With the gap detection unit, the voltage value was changed and the effect of voltage on the stability of the machining process was experimentally studied. The machining effect was evaluated using the statistical results of material removal rate and surface roughness, and the experimental results are shown in Figure 17 and Figure 18.

When a short-circuit occurred, the short-circuit state of the machining circuit was simultaneously with power failure and tool electrode retraction, so that continuous machining could be realized. From the above experiments, it was clear that the gap short-circuit detection method was still very effective in ensuring the continuity of the composite electric machining system, even as the feed rate increased during the machining process. Without gap protection, the machining process can be easily short-circuited under different voltage conditions, resulting in the machining process being forced to stop. The machining process continued with short-circuit detection protection, and the machining results are shown in the figure. As the processing voltage increased, the processing current density increased, the workpiece dissolution rate increased, and the material removal rate gradually increased to 1.35 mm^3^/min. However, as the voltage value increased, the stray corrosion phenomenon became more serious, the processing quality decreased, the electrolytic corrosion energy per time unit increased, and the surface roughness decreased by about 12%. In order to obtain a high material removal rate of the composite material, lower speed, higher voltage and a two-dimensional amplitude were required. When the voltage was 7 V, a spark discharge occurred during the machining process, and although the machining process was not interrupted by short-circuiting under the control system, the machining accuracy was significantly reduced. The reason for the spark discharge was that when the machining gap became smaller and smaller, the local point’s field strength was large enough to encounter a bump which was difficult to machine, and the contact formed a discharge channel, while the gap produced electrolytic hydrogen bubbles to reduce the electrical conductivity, which generated a spark discharge.

### 5.2. Verification Experiments for Optimal Selection of Machining Parameters

**Figure 19:**
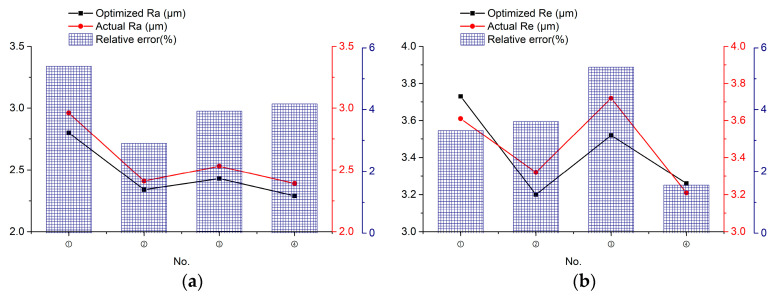

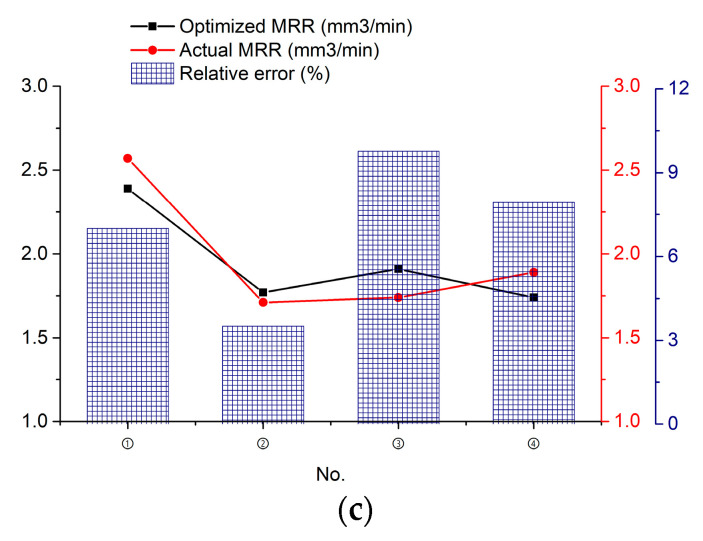
Distribution of different sample machining results: (**a**) $R_{a}$; (**b**) $R_{e}$; (**c**) MRR.

**Figure 20:**
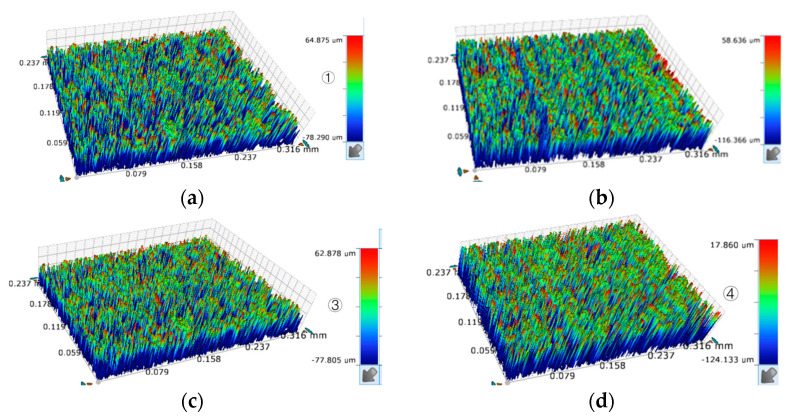
Micro machining morphology of different samples: (**a**) sample 1; (**b**) sample 2; (**c**) sample 3; (**d**) sample 4.

In order to check the accuracy and effectiveness of the proposed multi-objective method of optimization, any four groups of three-objective process optimization machining parameters were selected for experimental confirmation in Table 7, in which the relevant settings, steps, methods and testing instruments are the same as in the previous section. The relevant results are listed in the table, and the listed parameter combinations were all non-dominated solutions; in other words, they were not superior or inferior to each other, so any set of them represented an optimized solution. The mean values of surface roughness, material removal rate and edge accuracy of the four group samples were 2.54 μm, 1.92 mm^3^/min, 3.42 μm, respectively which are shown in Figure 19. Since the optimization results were converged, the differences between these optimized solutions were minor. With the selected combination of optimized machining parameters for combined machining experiments, comparisons between the experimental results and the optimized results are shown in Table 8, and the microscopic shape of the machined workpiece is shown in Figure 20.

From the experimental results, it can be seen that with the combination of process parameters optimized using the multi-objective optimization genetic algorithm, the actual processing results of the processing material removal rate and roughness processing index were very close to the multi-objective optimization results; in other words, the optimization results can truly reflect the change law of the process index with the value of process parameters.

**Figure 21:**
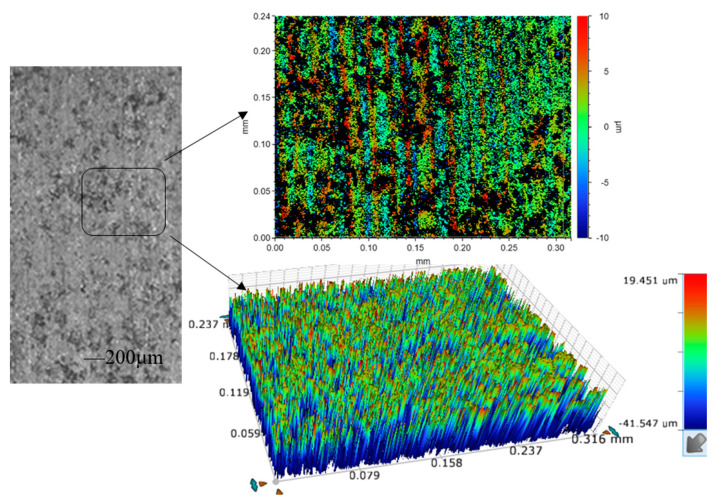
Partial machining of surfaces with optimal parameters.

When combining this model with the previous theoretical model, although the ultrasonics milled the particles and reduced the bump when increasing the voltage, the electrolysis/discharge processing effect was significant at higher voltages, while electrolysis/discharge reduced the height of the bump. However, the secondary processing by means of electrolysis increased the number of holes and the exposure depth of the residual particles after the particles were dislodged, thus increasing the roughness value. As a result, smaller amplitudes were configured for lower feed rates, while amplitudes were increased for higher feed rates, and voltages should be lower to reduce the hole effect of electrolysis on the enhanced particles. The Pareto optimal solutions with maximum satisfaction were selected as the optimal solutions to the multi-objective optimization problem, and the machining process parameters were obtained using the optimized process parameters. The maximum relative error of the obtained maximum machining surface roughness was 5.9%, the maximum relative error of the material removal rate was 5.5%, and the maximum relative error of the edge accuracy error was 8.9%. The traditional gray correlation analysis method was used to conduct a comparative experiment of the results of the multi-scale optimization of process parameters, and the results of the orthogonal machining experiments provided in this section were subjected to gray correlation analysis, where the voltage was 4.4 V, the spindle speed was 4400 rpm, the feed rate was 10.1 mm/min and the ultrasonic amplitude was 4.5 μm in both directions, which was set as the optimal solution of the multi-objective optimization problem, and the machining experiments were carried out with the above parameters for actual machining. The obtained surface roughness was 2.2 μm, the edge accuracy error was 3.4 μm, and the material removal rate was 1.6 mm^3^/min, as shown in Figure 21. This suggested that the multi-objective optimization genetic algorithm proposed in this chapter obtained a better combination of machining parameters. In addition, unlike the traditional gray correlation analysis method, which can only obtain a set of optimized solutions, the multi-objective genetic algorithm can obtain multiple optimal solutions, meaning that the machining process can have a wider range of machining parameters that can be selected according to the actual situation, and can thus meet different machining requirements.

The reasons for the errors in the optimized model were evaluated. On the one hand, the samples were not substantial enough, while other factors in the simplified model had less influence; in addition, the random influence in the machining process may have led to the discrepancy between the predicted and the actual machining effects. Overall, based on the above analysis, the experimental results can confirm the efficiency of the established combined electric machining parameter selection model. The intelligence of parameter selection can reduce the dependence on the operator’s experience, which can effectively improve the total performance of the machining system.

## 6. Conclusions

In order to solve the machining problem of difficult-to-machine materials, the 2DRUEM method was proposed in this study, and the machining gap and machining parameters optimization issues were also studied. The following conclusions were drawn based on the experimental results:

(1) Two-dimensional ultrasonic vibration promoted the renewal of the working fluid and the expulsion of machining products, which increased the discharge probability and frequency, and together with the movement of the abrasive grains in the grinding process, the surface activation of the workpiece was improved, and further increased the surface leveling effect of the workpiece, with a 20.1% increase in the material removal rate.

(2) With the characteristics of the combined machining system and the actual needs, a fusion scheme for detecting gaps using the gap current and grinding force ratio was adopted to determine machining short-circuits, and comparative experiments were conducted. These experiments showed that the method for short-circuit judgment and control by detecting the gap can effectively avoid short-circuit burns during machining and achieve continuous and stable machining, thus improving the machining efficiency and the surface quality of the workpiece. Moreover, for the fusion algorithm based on the PCA and the SVM for prediction, a gap error of 12.08% and a relative average error of 5.61% were obtained, which validated the usability of the fusion model scheme to some extent.

(3) Analyzing the parameters of the machining process, for the material removal rate, the machining tool ultrasonic amplitude and spindle speed were the main factors, followed by voltage; therefore, increasing speed and voltage can help to improve the machining efficiency. Meanwhile, for the surface roughness, spindle speed and workpiece amplitude were the main factors which could determine the surface quality of the machined workpiece. Additionally, the workpiece amplitude has a dominant effect on the edge roughness, followed by the tool amplitude. The triple-objective optimization of the machining parameters was performed, and a mathematical model of machining process parameter selection based on the principles of minimum roughness and material removal rate maximization was established for combined machining. The process parameters were optimized by the non-dominated ranking genetic algorithm, and a machining parameter optimization model based on NSGA-Ⅱ was established for the combined machining process. The Pareto optimal solution with maximum satisfaction was selected for machining experiments, and the obtained surface roughness was 2.2 μm, the edge accuracy error was 3.4 μm, and material removal rate was 1.6 mm^3^/min, which can validate the effectiveness and engineering application value of the optimization model.

## Figures and Tables

**Table 1:** Main parameters of orthogonal experimental machining.

Factors	Levels
Voltage U (V)	2, 3, 4, 5
Spindle speed $v_{s}$ (rpm)	1000, 3000, 4000, 5000
Feed rate $v_{w}$ (mm/min)	20, 40, 60, 100
Axial ultrasonic amplitude $A_{z}$ (μm)	1, 3, 4, 5
Radial ultrasonic amplitude $A_{x}$ (μm)	1, 3, 4, 5

**Table 2:** Results of orthogonal experiments.

No.	U (V)	$A_{z}\;(\mu m)$	$A_{x}\;(\mu m)$	$v_{s}\;(\mathrm{rpm})$	$v_{w}\;(\mathrm{mm}/\min)$	$R_{a}\;(\mu m)$	$R_{e}\;(\mu m)$	MRR (mm^3^/min)
1	2	1	1	1000	20	5.97	6.77	1.25
2	2	3	3	3000	40	4.25	4.93	3.16
3	2	4	4	4000	60	3.98	4.67	3.89
4	2	5	5	5000	100	4.27	4.60	4.51
5	3	1	3	4000	100	6.85	7.04	3.45
6	3	3	1	5000	60	3.07	6.72	2.89
7	3	4	5	1000	40	4.54	4.54	2.87
8	3	5	4	3000	20	3.08	4.12	3.84
9	4	1	4	5000	40	3.26	6.13	3.41
10	4	3	5	4000	20	3.52	4.46	4.97
11	4	4	1	3000	100	4.45	7.03	3.21
12	4	5	3	1000	60	4.95	5.27	2.96
13	5	1	5	3000	60	5.97	6.41	3.85
14	5	3	4	1000	100	5.70	5.99	4.35
15	5	4	3	5000	20	2.50	4.81	4.73
16	5	5	1	4000	40	4.35	5.26	2.58

**Table 3:** Relative factor effect of MRR, $R_{a}$, $R_{e}$.

Indicator Parameters	Input Parameters	Relative Factor Effect (%)
$R_{a}$	U	8.29
	$A_{z}$	19.67
	$A_{x}$	20.07
	$v_{s}$	29.38
	$v_{w}$	22.59
$R_{e}$	U	3.83
	$A_{z}$	29.30
	$A_{x}$	47.69
	$v_{s}$	4.72
	$v_{w}$	14.47
MRR	U	18.63
	$A_{z}$	13.32
	$A_{x}$	34.64
	$v_{s}$	28.36
	$v_{w}$	5.04

**Table 4:** Machining parameters’ range.

	U (V)	$A_{z}\;(\mu m)$	$A_{x}\;(\mu m)$	$v_{s}\;(\mathrm{rpm})$	$v_{w}\;(\mathrm{mm}/\min)$
Maximum value	2	1	1	1000	10
Minimum value	5	5	5	5000	100

**Table 5:** Some of the 3-objective process optimization machining parameters.

No.	U (V)	$A_{z}\;(\mu m)$	$A_{x}\;(\mu m)$	$v_{s}\;(\mathrm{rpm})$	$v_{w}\;(\mathrm{mm}/\min)$	$R_{a}\;(\mu m)$	$R_{e}\;(\mu m)$	$MRR\;({\mathrm{mm}}^{3}/\min)$
1	2.0	2.0	3.0	2003	14	3.13	4.38	2.63
2	5.0	5.0	3.4	4441	10	2.18	3.05	1.57
3	2.0	3.5	3.0	3135	11	2.68	3.84	2.24
4	3.1	4.7	3.1	4083	10	2.36	3.36	1.85
5	3.0	3.9	3.1	3853	10	2.44	3.49	1.92
6	2.0	2.8	3.0	2014	10	2.92	3.99	2.50
7	2.0	3.3	3.0	2007	11	2.88	3.88	2.45
8	3.2	4.4	3.1	2547	11	2.55	3.40	2.01
9	2.6	4.5	3.1	2598	11	2.61	3.54	2.12
10	3.2	4.3	3.1	3592	11	2.42	3.38	1.88
11	2.2	3.4	3.1	2840	10	2.68	3.77	2.22
	……							

**Table 6:** Basic experimental parameters.

Items	Parameters
Workpieces	SiCp/Al, 60%.
Tool electrodes	Diamond: diameter, 6 mm; grain density, 400 mesh.
Voltage	2~8 V.
Ultrasonic amplitude	Axial ultrasonic amplitude, 5 μm; radial ultrasonic amplitude, 5 μm.
Feed rate	10~50 mm/min.
Spindle speed	2000~5000 r/min.
Electrolyte	NaNO_3_, mass fraction, 5%.

**Table 7:** Optimization of machining parameters.

No.	U (V)	$A_{z}\;(\mu m)$	$A_{x}\;(\mu m)$	$v_{s}\;(\mathrm{rpm})$	$v_{w}\;(\mathrm{mm}/\min)$
①	4.1	3.0	2.0	2024.9	10.3
②	4.4	3.1	4.2	3445.3	10.2
③	3.1	3.8	3.5	4093.7	10.4
④	4.5	3.2	3.7	4408.9	10.2

**Table 8:** Comparison of experimental results.

Samples	Optimization Results			Actual Results		
No.	$R_{a}\;(\mu m)$	$R_{e}\;(\mu m)$	$MRR\;({\mathrm{mm}}^{3}/\min)$	$R_{a}\;(\mu m)$	$R_{e}\;(\mu m)$	$MRR\;({\mathrm{mm}}^{3}/\min)$
①	2.80	3.73	2.39	2.96	3.61	2.57
②	2.34	3.20	1.77	2.41	3.32	1.71
③	2.43	3.52	1.91	2.53	3.72	1.74
④	2.29	3.26	1.74	2.39	3.21	1.89

## Data Availability

The authors confirm that the data supporting the findings of this study are available within the article.
